# Ecological niche model of *Phlebotomus alexandri *and *P. papatasi *(Diptera: Psychodidae) in the Middle East

**DOI:** 10.1186/1476-072X-9-2

**Published:** 2010-01-21

**Authors:** Michelle G Colacicco-Mayhugh, Penny M Masuoka, John P Grieco

**Affiliations:** 1 Department of Sand Fly Biology, Division of Entomology, Walter Reed Army Institute of Research, 503 Robert Grant Avenue, Silver Spring, MD 20910, USA; 2 Uniformed Services University of the Health Sciences, Department of Preventive Medicine and Biometrics, 4301 Jones Bridge Road, Bethesda, MD 20814, USA

## Abstract

**Background:**

The purpose of this study is to create distribution models of two sand fly species, *Phlebotomus papatasi *(Scopoli) and *P. alexandri *(Sinton), across the Middle East. *Phlebotomus alexandri *is a vector of visceral leishmaniasis, while *P. papatasi *is a vector of cutaneous leishmaniasis and sand fly fever. Collection records were obtained from literature reports from 1950 through 2007 and unpublished field collection records. Environmental layers considered in the model were elevation, precipitation, land cover, and WorldClim bioclimatic variables. Models were evaluated using the threshold-independent area under the curve (AUC) receiver operating characteristic analysis and the threshold-dependent minimum training presence.

**Results:**

For both species, land cover was the most influential environmental layer in model development. The bioclimatic and elevation variables all contributed to model development; however, none influenced the model as strongly as land cover.

**Conclusion:**

While not perfect representations of the absolute distribution of *P. papatasi *and *P. alexandri*, these models indicate areas with a higher probability of presence of these species. This information could be used to help guide future research efforts into the ecology of these species and epidemiology of the pathogens that they transmit.

## Introduction

*Phlebotomus (Phlebotomus) papatasi *(Scopoli) and *P. (Paraphlebotomus) alexandri *(Sinton) are widely distributed across parts of Europe, Africa, and Asia. *Phlebotomus papatasi *is a vector of sand fly fever virus and *Leishmania major*, which causes cutaneous leishmaniasis [1-3]. *Phlebotomus alexandri *is a vector of *L. donovani *and is a suspected vector of *L. infantum*, both of which cause visceral leishmaniasis [4-6]. Though these species are important disease vectors, little is known about the ecology and distribution of each.

*Phlebotomus papatasi *ranges from Morocco and Spain, across the Mediterranean Basin to India and south to parts of the Sudan and Ethiopia [7]. *Phlebotomus papatasi *is most abundant in areas with a mean minimum temperature of 16°C and mean maximum temperature of 44°C from May to October [8]. It can be found at elevations ranging from near sea level to over 1100 m [9].

*Phlebotomus alexandri *ranges from Spain and Morocco east to the mountains in northwestern China and as far south as southern Ethiopia [10]. This species has been recorded at elevations ranging from sea level to 1500 m above sea level [11,12]. In Djibouti, this species is found on the coastal plain, inland plateau, and highland valleys [13].

Characterizing the distribution and ecology of these vector species would be valuable in better understanding the epidemiology of sand fly fever and leishmaniasis. Cross et al. (1996) developed a model of *P. papatasi *distribution in Southwest Asia based on weather and the normalized difference vegetation index (NDVI); however, the model was not validated. Since the study was completed, powerful presence-only modeling techniques and software have been developed.

These newer modeling methods include ecological niche modeling (ENM). ENM uses presence data in conjunction with environmental data to develop models of habitat range for a given organism [14]. It is often used to examine the distribution of species that have not had intense, methodological sampling. This technique has been used in modeling distribution of diseases, such as dengue, and vectors, such as *Anopheles gambiae *[15-19]. In addition, niche modeling has been used to examine the distribution and potential distribution of *Lutzomyia *spp. vectors of leishmaniasis in South America [15,16,20].

In this study, we use ENM to develop distribution models for *P. papatasi *and *P. alexandri *in the Middle East. Using these models, we attempt to identify environmental factors which influence the distribution of these species.

## Results

### Phlebotomus papatasi

**Figure 1:**
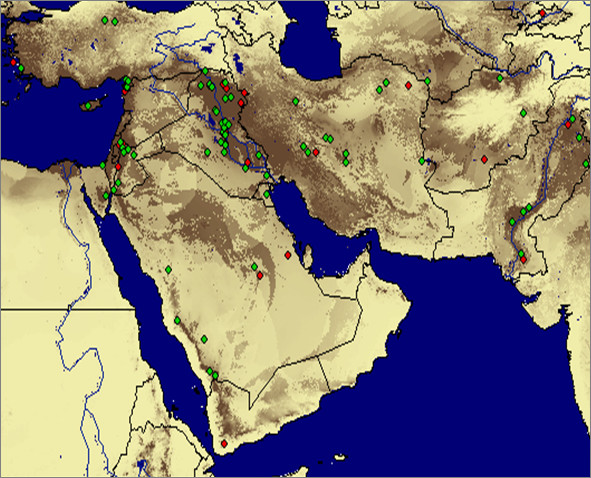
**Predicted distribution of *Phlebotomus pap *atasi in the Middle East**. Lighter areas indicate low probability of occurrence, darker areas indicate high probability of occurrence. Green points indicate training records and red points indicate test records.

The MaxEnt model for *P. papatasi *is shown in Figure 1. The AUC for the training points was 0.944 and for test points was 0.884, with a standard deviation of 0.042. The minimum training presence for a training point was 0.197; therefore, this was set as the threshold for binomial conversion. The fractional predicted area (the area coded as 1 = present) is 0.346 and the omission rate for test points was 0.091. At this threshold, the test points were classified significantly better by the model than by random selection (p < 0.0001).

**Figure 2:**
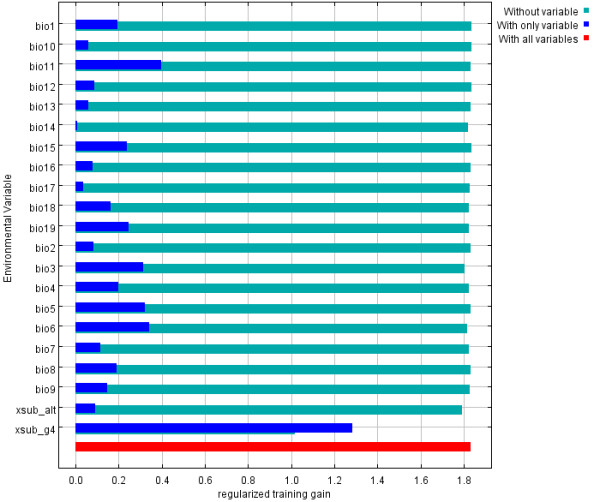
**Jackknife test of training gain for *P. papatasi***. Environmental variables: bio1through bio 19 represent the bioclimatic variables (Table 2); xsub_alt is the elevation layer; xsub_g4 is the land cover layer.

Jackknife tests of variable importance show that land cover was the most influential variable in model development (Figure 2). The training gain when land cover was the only variable used in model development was high, indicating that it contributes strongly to the model. When land cover was removed from the model, training gain dropped. This indicates that the land cover variable contains unique information that is required for model creation. The land cover types and probabilities associated with the training points are given in Table 1. Points classified as urban, field/woody savanna, and woody savannah coverages have high probabilities of presence. However, the sample size is small for both field/woody savanna and woody savanna. Points classified as bare desert have low probabilities of presence; however the sample size is small. All other classes have either very wide ranges or sample size of one.

**Table 1:** Land cover classes associated with training points for *Phlebotomus papatasi *and *P. alexandri*.

	**Probability of Presence**					
	
	***Phlebotomus papatasi***			***Phlebotomus alexandri***		
	
**Land Cover Class**	n*	Mean**	Range***	**n**	**Mean**	**Range**
Bare desert	5	0.2614	0.1852 - 0.3473	8	0.5708	0.0894 - 0.9724
Crops and town	6	0.6965	0.1798 - 0.9587	--	--	--
Crops, grass, and shrub	--	--	--	1	0.6880	--
Dry woody scrub	--	--	--	1	0.9791	--
Field/woody savanna	5	0.7955	0.6827 - 0.9383	5	0.8509	0.7250 - 0.9066
Grass crops	6	0.6958	0.2110 - 0.9427	3	0.5313	0.4324 - 0.6470
Hot, irrigated cropland	1	0.6714	--	--	--	--
Irrigated grassland	1	0.3477	--	1	0.4683	--
Low sparse grassland	4	0.3754	0.1729 - 0.6176	1	0.2610	--
Semi-desert shrubs	18	0.5220	0.2258 - 0.7222	27	0.6913	0.2099 - 0.9042
Urban	20	0.9576	0.7012 - 0.9915	10	0.9667	0.8889 - 0.9933
Woody savanna	2	0.9278	0.9174 - 0.9382	2	0.9191	0.8883 - 0.9500

* n = the number of training points that occurred in a given land cover class

** Mean is the mean probability of the species being present among points that occur in a given land cover class

*** Range is the range of probability values associated with the points that occur in that land cover class

The remaining variables contributed much less to model development (Figure 2). Isothermality (Bio3), maximum temperature in the warmest month (Bio5), minimum temperature in the coldest month (Bio6), mean temperature in the coldest quarter (Bio11), precipitation seasonality (Bio15), and precipitation in the coldest quarter (Bio19) all had modest gains when run with only the variable in question. However, the model gain was minimally decreased by the exclusion of the variables from the analysis. This is a reflection of the correlated nature of the bioclimatic variables and an indication that none of these variables in isolation are overwhelmingly contributing to this model.

### Phlebotomus alexandri

**Figure 3:**
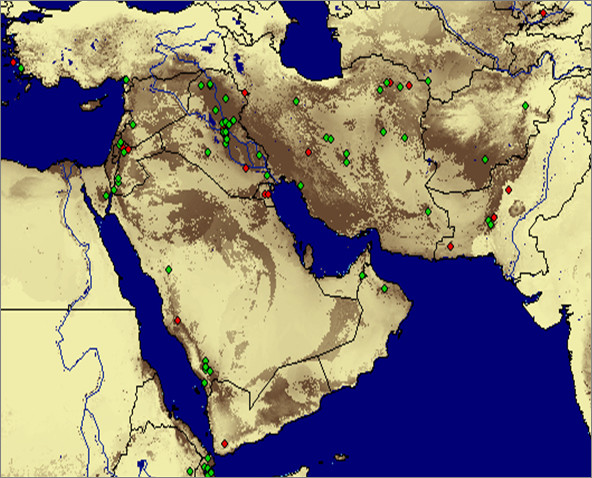
**Predicted distribution of *Phlebotomus alexandri *in the Middle East**. Lighter areas indicate low probability of occurrence, darker areas indicate high probability of occurrence. Green points indicate training records and red points indicate test records.

The MaxEnt model for *P. alexandri *is shown in Figure 3. The training AUC was 0.942 and the test AUC was 0.844, with a standard deviation of 0.044. The minimum training presence among training points was 0.164. At this threshold, the fractional predicted area was 0.368 and the omission rate for test points was 0.200. The model classifies the test points correctly significantly more than a random model (p < 0.0001).

**Figure 4:**
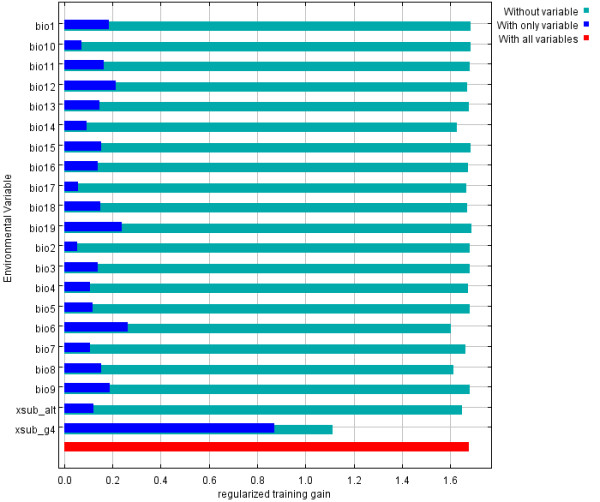
**Jackknife test of training gain for *P. alexandri***. Environmental variables: bio1through bio 19 represent the bioclimatic variables (Table 2); xsub_alt is the elevation layer; xsub_g4 is the land cover layer.

As in the model for *P. papatasi*, land cover was the most influential variable in modeling *P. alexandri *(Figure 4). Jackknife tests show high training gain when land cover is considered alone and a large drop training gain when land cover is omitted from the model. The land cover types and probabilities associated with the training points are given in Table 1. As with *P. papatasi*, points classified as urban, field/woody savanna, and woody savanna coverages have high probabilities of presence. However, the sample sizes for each of these habitats are small. All other classes have either very wide ranges or small sample size.

The 19 bioclimatic variables and elevation all have very modest training gains when considered in isolation (Figure 4), indicating that none of them strongly contribute to model development on their own. Elevation, minimum temperature in the coldest month (Bio6), mean temperature in the wettest quarter (Bio8), and precipitation in the driest month all show modest decreases in training gain when removed from the model. This indicates that they may contain unique information required for the model.

## Discussion

In this study, land cover contributed strongly to the development of the models for both species. Table 1 shows the different land cover classes associated with the species presence points that were used to develop the models. Though it is tempting draw direct conclusions from these data, one must view these results cautiously. The distribution of model development points in the different land cover classes simply reflects the locations of the points that were randomly selected as training points after the larger data set was entered into the modeling program. While these models may be used to drive hypotheses for further research into the ecology of *P. papatasi *and *P. alexandri*, they should not be taken as conclusive evidence of relationships between a certain land cover classification and the relative probability of species presence.

In the models presented here, the urban land cover class was associated with a high probability of presence for both species. This may be partly due to sampling bias, as collections of phlebotomine sand flies tend to be associated with research related to human leishmaniasis. However, in a survey of sand flies in Turkey, *P. alexandri *was present in the more urban areas of the province, but not in the rural areas [21]. *Phlebotomus alexandri *is anthropophilic. It is possible that this feeding relationship would drive the species to be more probable in urban environments simply because humans are more readily available. More ecological studies should be conducted to determine if there is a relationship between these species and urban areas or if this is a reflection of sampling bias toward collection of sand flies in areas where there is human disease.

Other non-urban land cover types are also important for these species. In the present study, the logistic probability of presence of both species for the points that fall in the woody savannah and field and woody savanna classes are high (mean 0.9278 and 0.7955, respectively), though the sample sizes are small. The range of probability of presence for both species in relation to semi-desert shrub is extremely wide for both species (Table 1), making it difficult to infer anything about this land cover class from these models. However, research in Morocco has shown that both species are associated with desert, scrub vegetation [22]. For *P. papatasi*, bare desert appears to be related to a low probability of presence in this study, though only five training points were located in this cover class. In Israel, *P. papatasi *is more abundant in areas with more humid soils, capable of supporting desert vegetation, than in areas with low soil moisture and less vegetation [23,24]. Sand flies require a sugar meal, taken from plant material [25,26]; therefore, they would be expected to be less abundant in areas with little or no vegetation, such as a barren desert. Further field-based ecological research is necessary to better determine what relationships exist between different land cover types and sand flies.

Cross et al. (1996) used normalized difference vegetation index (NDVI) and weather data to develop a predictive model of *P. papatasi *distribution in southwest Asia; however the models were not validated. We considered including 1-kilometer resolution NDVI data in this model; however, the addition of NDVI to the model development did not affect the outcome. When land cover was excluded and NDVI and the bioclimatic variables were used for model building, the resulting product did not perform as well as the models presented here. While NDVI may help model development in some cases, 1-km resolution NDVI data taken from Advanced Very High Resolution Radiometer (AVHRR) sensors was not as useful as other environmental and climatic variables in this case.

Temperature and precipitation are important for model development, but were not the leading factors in the development of the models presented here. In Morocco, sand flies are most active in the hot, dry season, with *P. papatasi *most abundant when ambient temperature is 32-36°C range [27]. In Pondicherry, India, *P. papatasi *reaches its peak abundance at the end of monsoon season [28]. Sand flies in Oman, particularly *P. alexandri*, are more abundant during periods of low humidity and high temperature (Roberts 1994). In the present study, the bioclimatic variables all contributed toward model development. However, none of these variables were particularly valuable in isolation for either model creation or validation. This is most likely a reflection of the correlated nature of the temperature and precipitation variables.

## Conclusion

These models are estimates of the distribution of *P. papatasi *and *P. alexandri*, based on the environmental layers chosen in the study and the location of collection records. As such they are not a definitive guide as to whether or not a species will be present in a given area. However, they can be used to estimate to the probability that *P. papatasi *and/or *P. alexandri *are present in an area. Since animal reservoir, *Leishmania *parasite, and/or leishmaniasis disease data are not included in these models, they do not reflect the distribution of disease. Future models incorporating records of *Leishmania *parasites, disease, and reservoir populations could provide a better understanding of the distribution and risk of cutaneous and visceral leishmaniasis in this area. Finally, further exploration of the relationship between different remote sensing products, such and land cover classifications and vegetations indices, and sand fly populations in the Middle East may help to further refine these models.

## Materials and methods

### Study Area

The study area includes part or all of the following countries: Afghanistan, Armenia, Azerbaijan, Cyprus, Egypt, India, Iran, Iraq, Israel, Jordan, Kuwait, Kyrgyzstan, Lebanon, Oman, Pakistan, Qatar, Saudi Arabia, Syria, Tajikistan, Turkey, Turkmenistan, the United Arab Emirates, Uzbekistan, and Yemen (Figure 1). The coordinates delineating the corners of the study area are: northwest corner, N 42.0819, E 25.4443; southwest corner, N 11.3508, E 25.443; northeast corner, N 42.0819, E 75.0586; southeast corner, N 11.3508, E 75.0586.

### Species Records

Presence data for the species were taken from records in the scientific literature dating from 1950 through 2007, collections performed by U.S. military entomologists in Iraq and Afghanistan between 2003 and 2006, and in Turkey in 2006. All coordinates were converted to the decimal degrees format.

The literature search was conducted using PubMed, searching on the terms "*Phlebotomus papatasi*," "*Phlebotomus alexandri*," "sand fly," and "sandfly." The search yielded 427 records for "*Phlebotomus papatasi*," 31 records for "*Phlebotomus alexandri*," 3026 records for "sand fly," and 3155 records for "sand fly." After exclusion of articles that did not include collection records of either species of interest and articles with records outside the region of interest, there were 31 publications for *P. alexandri *and 173 for *P. papatasi*. Records for which there was uncertainty about the location of the sampling or no specific location given were then excluded. This process contributed 98 *P. papatasi *presence points from 22 articles, by 20 first authors and 79 *P. alexandri *presence points from 25 publications, by 19 different first authors to the modeling process. For both species, the articles were published between 1968 and 2007.

Once the unpublished Afghanistan, Iraq, and Turkey collection data were added to the literature records, 115 points for *P. papatasi *and 98 points for *P. alexandri *were entered into the MaxEnt program for model development. The program was then set to exclude duplicate presence records within the same pixel. For *P. papatasi*, 25 points were excluded, leaving 90 presence records for model development and validation. Of these, 68 were randomly selected for model development, with the remaining 22 used to test the model. For P. alexandri, 18 records were excluded, leaving 80 presence records for model development and validation. Sixty of these records were randomly selected for model development, with the remaining 20 used to test the model.

### Environmental Layers

Climate and elevation layers were obtained from the WorldClim database, version 1.4 [29]. This database provides climate layers at a spatial resolution of 1 km^2 ^and is derived from weather station data from 1950-2000 [30]. For the purposes of this study, the WorldClim bioclimatic variables were used (Table 2).

**Table 2:** WorldClim bioclimatic variables used in model development.

Variable	Description of the Variable
Bio1	Annual mean temperature, C
Bio1	Mean diurnal range (mean of monthly (max temp - min temp)), C
Bio3	Isothermality ((Bio2.Bio7)*100), C
Bio4	Temperature Seasonality (standard deviation * 100), c
Bio5	Maximum temperature of warmest month, C
Bio6	Minimum temperature of coldest month, C
Bio7	Temperature annual range (Bio5 - Bio6), C
Bio8	Mean temperature of the wettest quarter, C
Bio9	Mean temperature of the driest quarter, C
Bio10	Mean temperature of the warmest quarter, C
Bio11	Mean temperature of the coldest quarter, C
Bio12	Annual precipitation, mm
Bio13	Precipitation of the wettest month, mm
Bio14	Precipitation of the driest month, mm
Bio15	Precipitation seasonality (coefficient of variation), mm
Bio16	Precipitation of the wettest quarter, mm
Bio17	Precipitation driest quarter, mm
Bio18	Precipitation of the warmest quarter, mm
Bio19	Precipitation of the coldest quarter, mm
Alt	Altitude (elevation above sea level), m

Land cover data were obtained from the U.S. Geological Survey's (USGS) Earth Resources Observation and Science (EROS) Data Center [31]. This is a global land cover classification that is broken into 96 land cover classes at 1-km resolution. Of the ninety-six classes, only 60 occur in the study area. The 68 training points for *P. papatasi *fall into just 10 of these classes (Table 1). The 60 training points for *P. alexandri *are associated with 10 classes (Table 1).

### Model Building and Evaluation

The niche modeling application MaxEnt was used in this analysis [32]. The MaxEnt program develops models of species distribution, subject to environmental variables entered into the model building process, using the principles of the maximum entropy distribution [33,34]. Models were developed using MaxEnt version 3.2.1.

Seventy-five percent of the data points for each species were randomly selected as training points, used in model building. The remaining 25% of the records were test points, used in model validation. Duplicate presence records were removed by the MaxEnt program prior to model development. The MaxEnt model output was set to logistic, which returns an estimated probability of presence for a given location between the values of 0 (no probability of species presence) and 1 (species is certain to be present). All other parameters were set to the default settings.

The model was evaluated using both threshold-dependent and threshold-independent methods. The area under the curve (AUC) of the receiver operating characteristic (ROC) analysis is a threshold-independent method of evaluating model quality. This technique computes the total area under the curve created by plotting sensitivity against the fractional predicted area for the species [33-35]. The threshold-dependent measure used here is the minimum training presence in which the probabilities are converted to binomial values with 0 being absent and 1 being present [33-35]. Using this method, all pixels with a probability of presence equal to or greater than that of the training point with the lowest probability of presence are classified as present and all pixels with a lower probability of presence are classified as absent. A one-tailed binomial test is then performed with the null hypothesis being that the model does not predict the test points better than random [33].

In order to determine which variables contribute most to the model development, the MaxEnt program was set to calculate jackknife tests of variable importance. The jackknife procedure produces three different types of models: (1) models created with one variable at a time excluded and all other variables included, (2) models created with only one variable included, and (3) a model created with all variables [33-35]. Variables that are most important to model development are those that decrease the training gain when removed from the model and show gain when the model is developed with only one variable.

## Competing interests

The authors declare that they have no competing interests.

## Disclaimer

Material has been reviewed by the Walter Reed Army Institute of Research. There is no objection to its publication. The opinions or assertions contained herein are the private views of the author, and are not to be construed as official, or as reflecting true views of the Department of the Army or the Department of Defense.

## Authors' contributions

MGC conceived and executed this study and drafted the manuscript. PMM obtained the remote sensing imagery used in the study and assisted in model development. JPG helped develop the concept of the study and provided resources for its execution. PMM and JPG assisted in the development of the final manuscript.
